# Effect of Nanocarbon on the Structural and Mechanical Properties of 6061 Aluminum Composites by Powder Metallurgy

**DOI:** 10.3390/nano13222917

**Published:** 2023-11-08

**Authors:** Wilson Rativa-Parada, Hansika I. Sirikumara, Robinson Karunanithy, Poopalasingam Sivakumar, Thushari Jayasekera, Sabrina Nilufar

**Affiliations:** 1 School of Mechanical, Aerospace, and Materials Engineering, Southern Illinois University, Carbondale, IL 62901, USA; 2 E. S. Witchger School of Engineering, Marian University, Indianapolis, IN 46222, USA; hsirikumara@marian.edu; 3 School of Physics and Applied Physics, Southern Illinois University, Carbondale, IL 62901, USA; robinson.karunanithy@siu.edu (R.K.); thushari@siu.edu (T.J.)

**Keywords:** nanocarbon–aluminum composites, graphene nanoplatelets, activated nanocarbon, micro-Vickers hardness

## Abstract

6061 aluminum composites with 0.5 and 1 vol. % graphene nanoplatelets as well as 1 and 2 vol. % activated nanocarbon were manufactured by a powder metallurgy method. Scanning electron microscopy and Raman spectroscopy were used to study the morphology, structure, and distribution of nanocarbon reinforcements in the composite samples. Density Functional Theory (DFT) calculations were performed to understand the aluminum-carbon bonding and the effects of hybridized networks of carbon atoms on nanocarbon aluminum matrix composites. Scanning electron microscopy showed the good distribution and low agglomeration tendencies of nanoparticles in the composites. The formation of secondary phases at the materials interface was not detected in the hot-pressed composites. Raman spectroscopy showed structural changes in the reinforced composites after the manufacturing process. The results from Density Functional Theory calculations suggest that it is thermodynamically possible to form carbon rings in the aluminum matrix, which may be responsible for the improved mechanical strength. Our results also suggest that these carbon networks are graphene-like, which also agrees with the Raman spectroscopy data. Micro-Vickers hardness and compressive tests were used to determine the mechanical properties of the samples. Composites presented enhanced hardness, yield and ultimate strength compared to the 6061 aluminum alloy with no nanocarbon reinforcement. Ductility was also affected, as shown by the reduction in elongation and by the number of dimples in the fractured surfaces of the materials.

## 1. Introduction

Aluminum matrix composites (AMCs) have been extensively investigated in the last few decades owing to the increasing demand for stronger and more lightweight materials for the automobile, electronic, and aerospace industries [1,2,3,4,5,6]. AMCs have been produced using different manufacturing processes such as powder metallurgy, spark plasma sintering, stir casting, friction stir processing, and severe plastic deformation [7,8]. However, several factors, such as the poor dispersion of reinforcement and weak interfacial bonding, can deteriorate the properties of these composites [9]. Powder metallurgy is a simple and low-cost method that has shown effectiveness in homogenously dispersing reinforcements in the aluminum matrix with enhanced mechanical properties [10,11]. Within the powder metallurgy process, ball milling has been widely utilized as the procedure to mix and disperse the reinforcement due to its simplicity and scalability. The properties of AMCs depend on the duration and speed of milling, as well as the sintering and consolidation of the composites. The influence of these parameters has been investigated in the last few years, showing that excessive ball milling can also alter the structures of the milled materials [12]. Particularly, the consolidation step influences the densification and the formation of undesirable phases, such as Al_4_C_3_, which is highly influenced by temperature and time [13].

Besides this, materials used as reinforcement play a key role in AMCs. Historically, silicon carbide (SiC) [14], aluminum oxide (Al_2_O_3_) [15], and titanium carbide (TiC) [16] have been the conventional materials used in AMCs. However, persistent issues related to weak interfacial bonding and poor dispersion limit their use. For this reason, some alternative reinforcements of AMCs have been investigated with remarkable findings. Recently, Fuse et al. utilized stir processing to form boron carbide (B_4_C)-6061 aluminum composites that presented the uniform dispersion of B_4_C with enhanced hardness and wear resistance [17]. Desai et al. [18] also obtained fly ash (0 to 25 wt. %)-reinforced AMCs with enhanced wear behavior, hardness, and tensile strength. Likewise, titanium diboride (TiB_2_) has been used in 6061 aluminum matrix composites. Pazhouhanfar et al. reinforced 6061 matrix composites with several amounts of TiB_2_ and tested their mechanical properties. The results showed that the ultimate strength rose as a function of the volume fraction of the reinforcement without significant loss of elongation, which was mainly attributed to load transfer, grain size reduction, and Orowan mechanisms [19]. Moreover, Chamroune et al. [20] employed graphite flakes as reinforcement in AMCs by powder metallurgy, which increased the anisotropic thermal properties due to the preferred orientation of the graphite flakes and deformations at the interface.

Within the variety of materials studied to reinforce AMCs, carbon allotropes have drawn the attention of researchers due to their excellent chemical, physical, and mechanical properties [21,22,23,24]. The most common allotropes of carbon include fullerene, nanodiamond, carbon nanotubes, graphene, and activated carbon. Graphene, a 2-dimensional arrangement of sp^2^ hybridized carbon with outstanding surface, electrical, thermal, and mechanical properties, has excelled as a reinforcement for AMCs [25,26,27,28,29]. The load transfer mechanism between aluminum and graphene is superior to that of aluminum with other sp^2^ hybridized carbons such as nanotubes and fullerenes, due to a better interface made of planar graphene and aluminum [30]. Nevertheless, graphene presents some challenges related to agglomeration and dispersion in the AMCs [31]. Activated carbon is another carbon allotrope with excellent surface properties [32] and has been employed in metal matrix composites as a source of carbides [33], graphene [34], and as a reinforcement for AMCs [35] with remarkable results.

This work focused on determining the influence of the synthesis parameters as well as the effects of the type of nanocarbon reinforcements, such as activated nanocarbon and graphene nanoplatelets, on the structure and performance of as-sintered 6061 aluminum matrix composites. The structural and mechanical properties were determined with Scanning Electron Microscopy (SEM), Raman Spectroscopy, Uniaxial Compression Testing, and micro-Vickers hardness. DFT calculations were performed to elucidate the atomic scale phenomena behind the carbon-aluminum interactions. The effects of the volume fraction of activated nanocarbon and graphene nanoplatelets on the behavior of the as-sintered AMCs were also discussed.

## 2. Materials and Methods

### 2.1. Composite Preparation

Both activated nanocarbon 6061 aluminum and graphene 6061 aluminum composites were fabricated by a powder metallurgy method. 6061 aluminum powders (Al: 95.8–98.6%. Chromium: 0.07%. Copper: 0.27%. Iron: 0.17%. Manganese: 0.01%. Magnesium: 0.86%. Silicon: 0.58%. Zirconium: <0.01%. Titanium: 0.02%. Others: <0.15%) were acquired from Valimet, Inc. (Stockton, CA, USA). At the same time, both nanocarbon powders, i.e., activated nanocarbon (<100 nm; 95%) and graphene nanoplatelets (99.5%), were acquired from US Research Nanomaterials, Inc. (Houston, TX, USA). A SPEX 8000 mixer from SPEX SamplePrep (Metuchen, NJ, USA) with a motor speed of 1450 rpm was utilized for milling the composites. Corresponding amounts of activated nanocarbon/graphene nanoplatelets and 6061 aluminum powders were mechanically mixed for 4 h within intervals of 30 min of milling with pauses of 30 min to maintain low-temperature conditions. This was done in a stainless steel jar using 5 mm steel balls in a 10:1 ratio and 1 wt. % of stearic acid (CH_3_(CH_2_)_16_COOH) from Sigma Aldrich (95%) as a lubricant agent. Before pressing, the stearic acid was evaporated for 30 min at 450 °C. Hot isostatic pressing was used to consolidate the previously milled powder composites with diverse volume fractions of nanocarbon reinforcement. The mixture was placed in a graphite die equipped with graphite punches of 25.4 mm in diameter and pressed for 1 h at 550 °C and 70 MPa under vacuum conditions in a Front-Loading Hot Press Furnace from Materials Research Furnaces LLC (Allenstown, NH, USA). The denomination of the final materials is shown in Table 1.

### 2.2. Structural Characterization

The surfaces of the as-sintered composite samples were ground with several abrasive papers (80, 120, 220, 320, 400, 600, 800, and 1200) and then polished with a soft cloth and a polishing suspension (sol-gel alumina). Both sintered and fractured morphologies were observed with a Quanta FEG 450 Scanning Electron Microscope equipped with energy-dispersive X-ray spectroscopy by Oxford Instruments (Concord, MA, USA). Samples were sputtered with an alloy of Au-Pt to enhance the definition of the images. The structures of the reinforcement materials were investigated using a Horiba iHR550 by Horiba Scientific (Irvine, CA, USA) imaging spectrometer in conjunction with a BX 41 microscope by Olympus (Center Valley, PA, USA) with 10×, 20×, 50×, and 100× magnification objectives and a near-infrared (NIR) excitation light source of 785 nm iBeam-Smalt-785-S-WS by TOPTICA Photonics (Pittsford, NY, USA). A grating of 600 gr/mm was used in the spectrometer. For the powder samples, a 10× object with 10 mW laser power was used to avoid burning the samples. For the compacted samples, the spectra were collected using the 10× objective at 120 mW laser power with a 15 s acquisition time and with a total of 10 scans over the desired range. In addition, bulk density was calculated by following the Archimedes principle.

### 2.3. Computational Calculations

Density Functional Theory calculations were carried out to understand the aluminum-carbon (Al-C) bonding and the effect of hybridized networks of C atoms in the Al matrix. Previous studies have investigated interfacial interactions between epitaxial graphene on the Al surface [36]. In this study, a network of C atoms sandwiched in a matrix of aluminum was considered. The model consists of a 2 × 2 × 4 Al structure and a C layer with 21 carbon atoms in the direction (100). The calculation cell has a dimension of 8.1 Å in the *x* and *y* directions and 18.8 Å in the *z* direction. In its pristine form, the 2 × 2 × 4 supercell of Al has a periodic length of 16.2 Å in the *z*-direction. All the atomic coordinates and the cell dimension in the z direction are elongated to insert the layer of carbon and the *z* dimension is fully optimized to minimize the force to 0.02 eV/Å. All energies were calculated using the ab initio Density Functional Theory implemented in the Quantum Espresso package [37]. The formation energy of the network of C atoms sandwiched in the Al matrix was evaluated by Equation (1):

(1) $$E_{f}=E_{AlC\;}-E_{Al}-E_{C}$$

where $E_{AlC}$ is the total energy of the Al/C system, $E_{Al}$ is the total energy of the Al matrix, and $E_{C}$ is the total energy of the C layer. Additionally, we calculated the phonon frequency of the Al/C system at the Gamma point, as it is implemented in the quantum espresso package.

### 2.4. Mechanical Testing

A micro-Vickers hardness tester HMV-G 31 DT from Shimadzu Scientific Instruments, Inc. (Pittsburgh, PA, USA) was used to obtain the hardness of the materials by following the ASTM E384 standard [38]. A load of 1.961 N was employed for all the measurements, and a total of 5 measurements per sample were taken at distinct locations using a square pyramidal indenter. Uniaxial compressive tests were performed according to the ASTM E9 standard [39] at room temperature in an MTS Insight 30 kN Standard Length testing machine employing rectangular samples with a 2:1 length/width ratio. A total of 3 tests were carried out for each composition. Yield strength was calculated by using the 0.2% offset method. Ultimate strength, modulus of toughness, and elongation were determined from average values obtained from the stress-strain diagrams.

## 3. Results and Discussion

### 3.1. Structural Characterization

**Figure 1:**
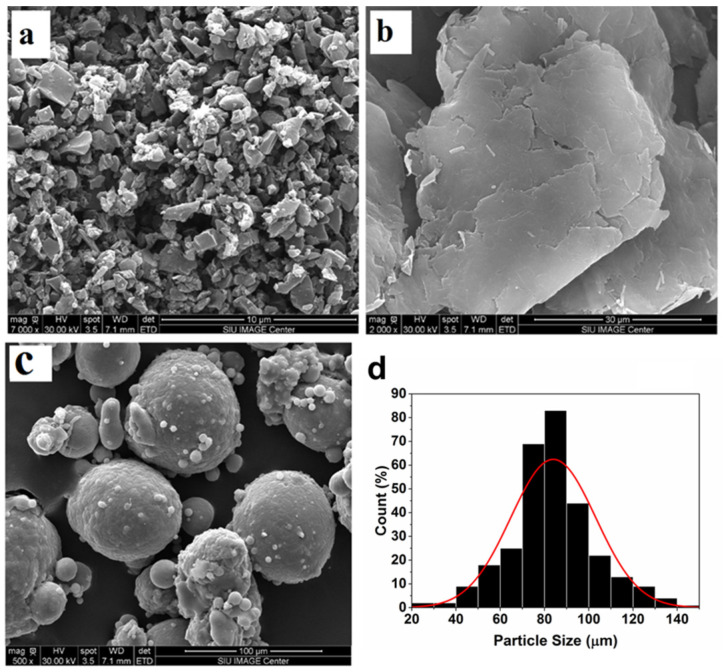
Morphology of the as-received (**a**) activated nanocarbon, (**b**) graphene nanoplatelets and (**c**) 6061 aluminum powders, and (**d**) size distribution of 6061 aluminum powders.

**Figure 2:**
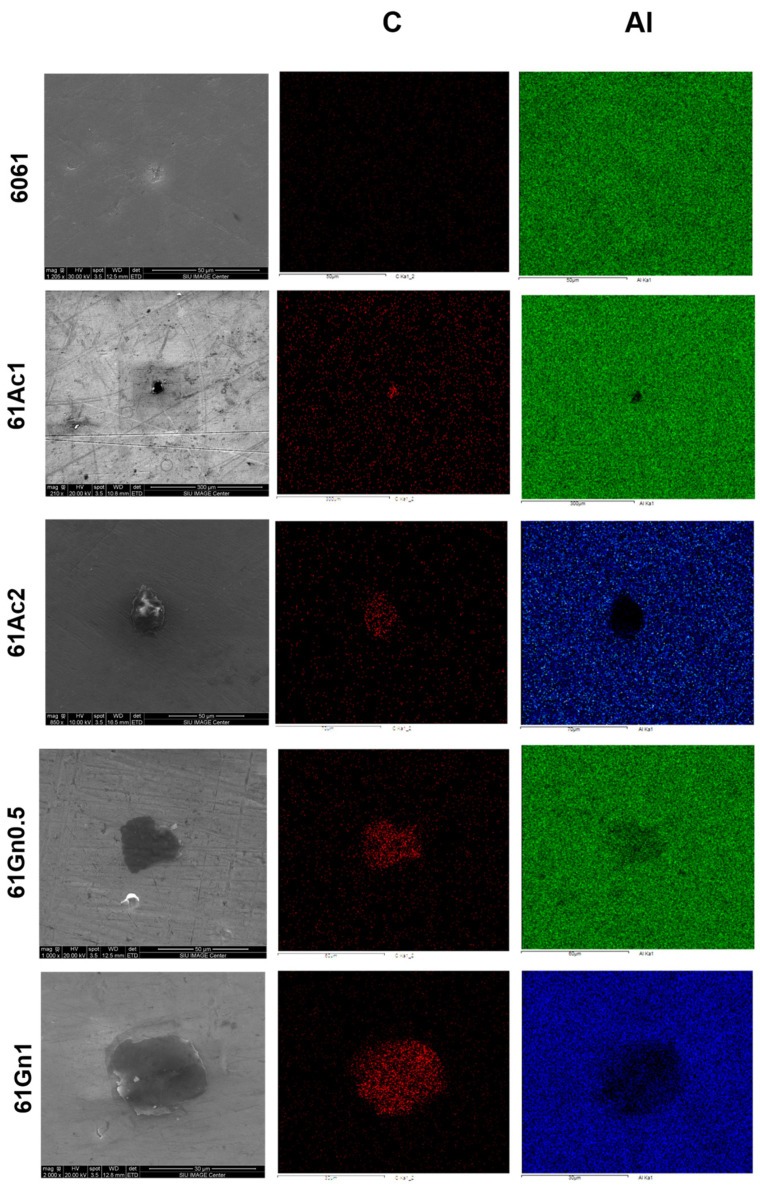
Morphology of the nanocarbon 6061 AMCs with EDS elemental mapping for aluminum (Al) and carbon (C).

Figure 1 displays the as-received powders of activated nanocarbon and graphene nanoplatelets, along with the morphology and size distribution of the 6061 aluminum alloy powders. The structure and distribution of activated nanocarbon and graphene nanoplatelets inside the as-sintered 6061 aluminum matrix with no evidence of a large-scale agglomeration are displayed in Figure 2, along with the EDS elemental mapping with the distribution for aluminum (Al) and carbon (C).

**Figure 3:**
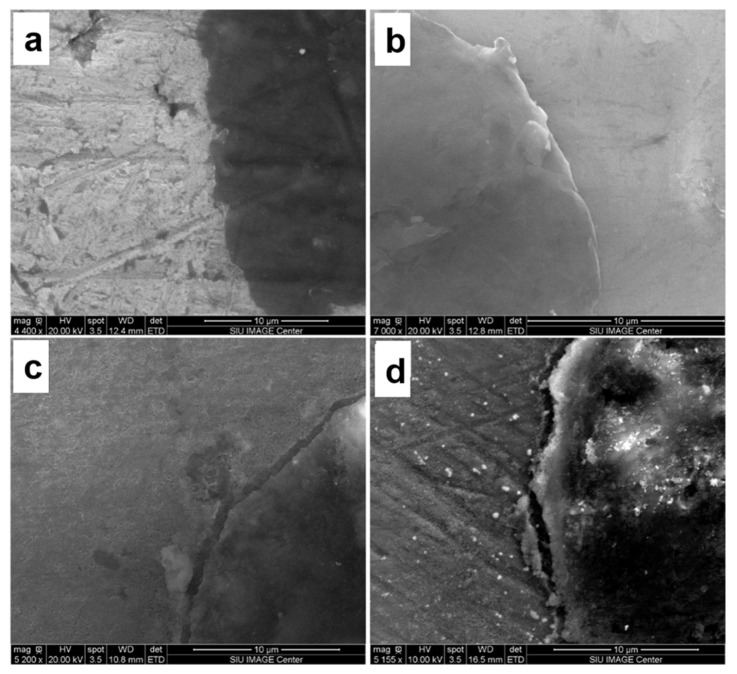
Nanocarbon-6061 aluminum interface of the nanocarbon 6061 aluminum composites (**a**) 61Gn0.5, (**b**) 61Gn1, (**c**) 61Ac1, and (**d**) 61Ac2.

Figure 3 presents the micrographs of the materials at the interface. According to SEM studies, the formation of intermediate phases such as Al_4_C_3_ at the carbon-aluminum interface during the synthesis of the composites was not evident. This suggests that the change in properties cannot be attributed to the inclusion of secondary phases at the interface but to the strengthening mechanisms between the matrix and reinforcement, including load transfer [40], thermal expansion mismatch [41], and Orowan Looping [42].

**Figure 4:**
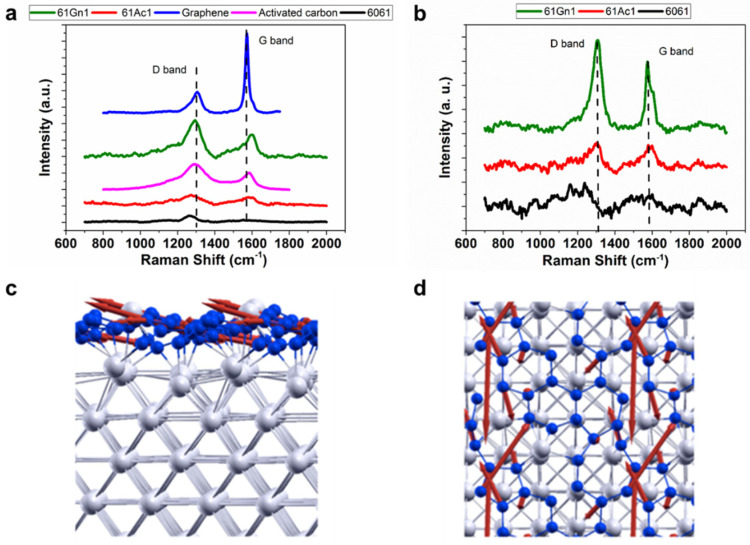
(**a**) Raman spectra of the nanocarbon 6061 AMCs after milling, (**b**) Raman spectra of the nanocarbon 6061 AMCs after compression, (**c**) side view, and (**d**) top view of atomic displacement pattern (shown in red) for the softest phonon frequency 1360 cm^−1^ for the atomistic model used for this calculation.

Raman characterization of the nanocarbon aluminum 6061 composites after milling and compression is shown in Figure 4a,b, and Table 2. The spectra for activated nanocarbon and graphene nanoplatelets display two peaks at 1350 and 1580 cm^−1^, corresponding to the D and G bands, respectively [43]. No peak corresponding to aluminum carbide (~850 cm^−1^ [44]) can be observed, which supports the SEM-EDS results shown above. The intensity of these peaks changes for the finalized composites compared to the initial materials, especially for the graphene nanoplatelets due to the structural changes suffered after the manufacturing process. This is confirmed by determining the intensity ratio between the D and G bands. The *I_D_*/*I_G_* ratio for as-received activated nanocarbon and graphene nanoplatelets are 1.50 and 0.26, respectively. After that, the *I_D_*/*I_G_* ratio changed to 1.65 and 1.27 for the milled activated nanocarbon and graphene, respectively, due to the increasing degree of disorder in the sp^2^ structures of the nanocarbon reinforcements [45]. This follows previous tendencies in which the increasing milling time accelerated the formation of defects and disorder in the graphene structure of reinforced 6061 aluminum composites [46]. Similarly, the *I_D_*/*I_G_* ratio continued decreasing after the compression procedure, although to a smaller degree. The crystallite sizes (*Cs*) for activated nanocarbon and graphene before and after the milling process are also determined with Equation (2) [47], showing that the crystallite sizes for both reinforcements increased with the increasing of defects in the structure, this value being higher for the activated nanocarbon-reinforced samples:

(2) $$Cs:\;\frac{{2.4\times 10}^{-10\;}\times {\lambda_{laser}}^{4}}{I_{D}/I_{G}}$$

**Table 2:** *I_D_*/*I_G_* ratio and crystallite size of the reinforcements in the nanocarbon 6061 AMCs after milling and compression procedures.

		Graphene Nanoplatelets	Activated Nanocarbon	61Ac1	61Gn1
Milled	I_D_/I_G_ ratio	0.26	1.50	1.65	1.67
	Crystallite Size (nm)	345.47	60.43	71.64	54.94
Compacted	I_D_/I_G_ ratio	-	-	1.11	1.33
	Crystallite Size (nm)	-	-	81.84	68.46

In addition to the microstructural analysis of the experimentally sintered composites, Density Functional Theory (DFT) calculations are shown in Figure 4c,d. There, the optimized structure, along with the atomic displacements corresponding to the highest phonon frequencies, is presented. When comparing the density of carbon atoms in pristine graphene, this model has 97% carbon coverage. This particular configuration has six-member carbon rings in the presence of defects. In a previous article, it was shown that the C rings in an Al matrix are energetically more favorable than isolated C atoms in an Al matrix [48]. The formation energy calculations show that the C layer in the Al matrix has a formation energy of −0.14 eV/atom, which shows that the sp^2^ hybridized C layer is thermodynamically stable. Phonon frequencies were calculated as implemented in the Quantum Espresso package. Non-negative frequencies for the gamma point phonons show the dynamical stability of the composite structure. The highest phonon frequency for C rings in aluminum is shown to be at 1360 cm^−1^, which is closer to the graphene peak observed in Raman spectroscopy, whereas isolated C scattered in Al shows 330 cm^−1^ of the highest phonon frequency. This result suggests the favorable formation of sp^2^ hybridized C rings in the Al matrix. In addition, the atomic displacement pattern presented in the figures shows a similarity to that of the D band in pristine carbon structures. The atomic displacement pattern also suggests that the C matrix vibrations are decoupled from the Al matrix, which is in agreement with the findings of previously reported studies on aluminum matrix composites [49]. Raman spectroscopy data confirm the existence of sp^2^ hybridized C rings in our samples. The results from DFT suggest that sp^2^-like carbon rings can be formed inside the aluminum matrix.

### 3.2. Mechanical Properties Characterization

**Figure 5:**
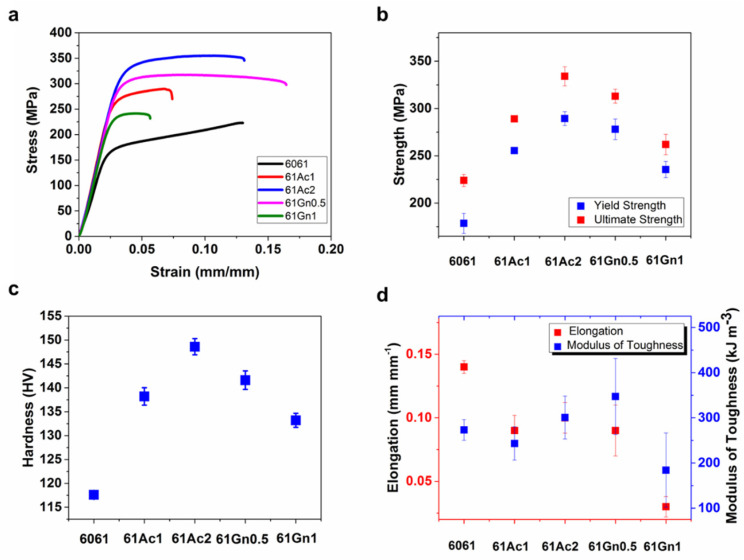
(**a**) Representative stress-strain diagrams for the compression tests of the nanocarbon 6061 AMCs. (**b**) Average Yield Strength and Ultimate Strength of the nanocarbon 6061 AMCs. (**c**) Average Hardness of the nanocarbon 6061 AMCs. (**d**) Average Modulus of Toughness and Elongation of the nanocarbon 6061 AMCs.

The mechanical properties of the AMCs were also determined experimentally through the uniaxial compression test and micro-Vickers hardness test. Experimental mechanical properties of the composites with and without nanocarbon reinforcements are summarized in Table 3. Representative compression test curves for each composite and average results for mechanical properties appear in Figure 5a and Figure 5b–d, respectively. The composites reached up to 61% and 55% increments of yield strength and up to 49% and 39% increments of ultimate strength for the activated nanocarbon and graphene reinforcements, respectively. However, yield and ultimate strength showed a decrease with the increasing graphene volume fraction due to the increased agglomeration of this carbon allotrope.

Conversely, both yield and ultimate strengths increased with the increasing volume fraction of activated nanocarbon. This behavior is attributed to a stronger dispersion and interfacial bond between the activated nanocarbon and aluminum matrix. The strengthening of the composites can be ascribed to the load transfer from the matrix to the stiffer reinforcement [50], as well as to the limited dislocation motion of aluminum [51]. Likewise, elongation and modulus of toughness were affected by the limited motion of dislocations, propitiated by the presence of the carbonaceous reinforcements, particularly for the samples with graphene, because of the tendency of graphene for localized agglomeration at the grain boundaries of the metal matrix [52,53]. However, in the case of the activated nanocarbon-reinforced samples, after the initial reduction, the values of elongation and modulus of toughness started to increase with the amount of reinforcement, which could propitiate a re-establishment of these properties at higher activated nanocarbon volume fractions. Manufacturing parameters also affect the mechanical properties of the composites, higher sintering and consolidation temperatures will produce stronger and harder samples. For example, Gürbüz et al. manufactured graphene/aluminum matrix composites at 550, 600, and 630 °C. The results show that the composites obtained at 630 °C presented enhanced apparent density and hardness compared to those fabricated at lower temperatures [54]. However, higher temperatures can also produce negative results, such as the formation of undesirable amounts of secondary phases (Al_4_C_3_), which will reduce the mechanical properties [55,56]. The introduction of activated nanocarbon and graphene nanoplatelets inside the metal matrix increased the hardness of the materials, with a maximum increment of 26% for the sample with 2 vol. % of activated nanocarbon, compared to the non-reinforced 6061 alloy. Reinforcement with these specific nanocarbon composites showed a similar behavior compared with other carbon allotropes such as C60, wherein the reinforcement with this allotrope increased the hardness of the composites up to 26% [57]. This increase in hardness has been attributed to good densification and a more limited deformation produced for activated nanocarbon and graphene in the metal matrix, as well as an effective refinement of the grains [45,58]. These results confirm previous reports wherein the increase in volume fraction of graphene nanoplatelets after 1 vol % had an unfavorable effect on the hardness due to the formation of vacancies and agglomeration [59]. Also, a larger presence of porosity has been associated with a decrease in the hardness of the composites [60].

**Table 3:** Mechanical properties of the nanocarbon 6061 AMCs.

Sample	Hardness (HV)	% of Increment	Yield Strength (MPa)	% of Increment	Ultimate Strength (MPa)	% of Increment	Elongation (mm mm^−1^)	% of Increment	Modulus of Toughness (kJ m^−3^)	% of Increment
6061	117.6 ± 2	-	178.6 ± 10	-	224 ± 6	-	0.14 ± 0.005	-	273.1 ± 23	-
61Ac1	138.2 ± 1	17.5	255.6 ± 2	43.0	289 ± 2	28.8	0.09 ± 0.01	−33.3	243.1 ± 36	−10.9
61Ac2	148.6 ± 1	26.3	289.3 ± 7	61.9	334 ± 10	49.1	0.10 ± 00.01	−23.8	300.7 ± 47	10.11
61Gn0.5	141.6 ± 1	20.4	278 ± 10	55.5	313 ± 7	39.8	0.09 ± 0.02	−30.9	347.2 ± 84	27.11
61Gn1	133.2 ± 0.8	13.2	235.5 ± 8	31.8	262 ± 10	17.1	0.03 ± 0.008	−73.8	184.1 ± 82	−32.5

**Figure 6:**
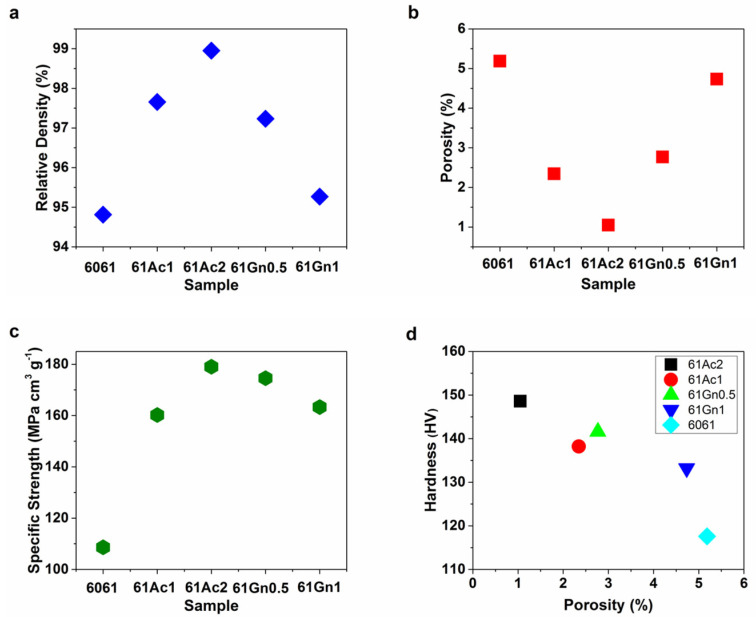
(**a**) Relative Density, (**b**) Porosity, (**c**) Specific Strength, and (**d**) Hardness as a function of porosity of the nanocarbon 6061 AMCs.

Results for the densities of the composites obtained experimentally are displayed in Figure 6a–d and in Table 4. All the samples presented a lower density, following the tendency presented by the theoretical values. Theoretical density was calculated using the rule of mixtures (3):

(3) $$\rho_{c}=V_{m}\rho_{m}+V_{r}\rho_{r}$$

where *ρ_m_* and *ρ_r_* are the density for matrix and reinforcement, respectively. Similarly, *V_m_* and *V_r_* are the volume fraction of the matrix and reinforcement, respectively.

Porosity was determined using Equation (4). The low level of porosity reflects the high degree of distribution reached using the powder metallurgy method [61].

(4) $$Porosity\;\left(\%\right)=\frac{\rho_{t}-\rho_{e}}{\rho_{t}}\times 100\%$$

where *ρ_t_* and *ρ_e_* are the theoretical and experimental densities, respectively.

All the compositions presented high relative densities (%), with a maximum of 98% for the sample with the higher volume fraction of activated nanocarbon due to better packaging and mechanical bonding compared to the other compositions, as also shown previously for aluminum matrix composites sintered with similar experimental methodology [62]. Figure 6d shows how the change in porosity is related to the hardness of the materials, showing that the materials with the highest levels of porosity present the lowest hardness, which is associated with gas entrapment during the manufacturing process [63], and the increasing agglomeration of the reinforcement [64]. Porosity has been linked to other mechanical properties of the aluminum matrix composites. Samples consolidated at higher compressive forces have presented lower levels of porosity and higher compressive strength [65]. Likewise, the increase in activated nanocarbon favored both the increase in hardness and decrease in porosity, unlike the case of graphene reinforcement, where the increase in volume fraction generated higher agglomeration, which is in agreement with the results in the literature [66]. These results of density differ from other conventionally used reinforcements, such as SiC [67] and TiC [68], whose densities increased with the reinforcement volume fractions.

**Table 4:** Theoretical and experimental density-related properties for the nanocarbon 6061 AMCs.

	Sample				
	6061	61Ac1	61Ac2	61Gn0.5	61Gn1
Experimental Density (g cm^−3^)	2.56	2.61	2.62	2.62	2.56
Theoretical Density (g cm^−3^)	2.70	2.67	2.65	2.69	2.69
Relative Density (%)	94.8	97.6	98.9	97.2	95.2
Porosity (%)	5.18	2.34	1.04	2.76	4.73

**Figure 7:**
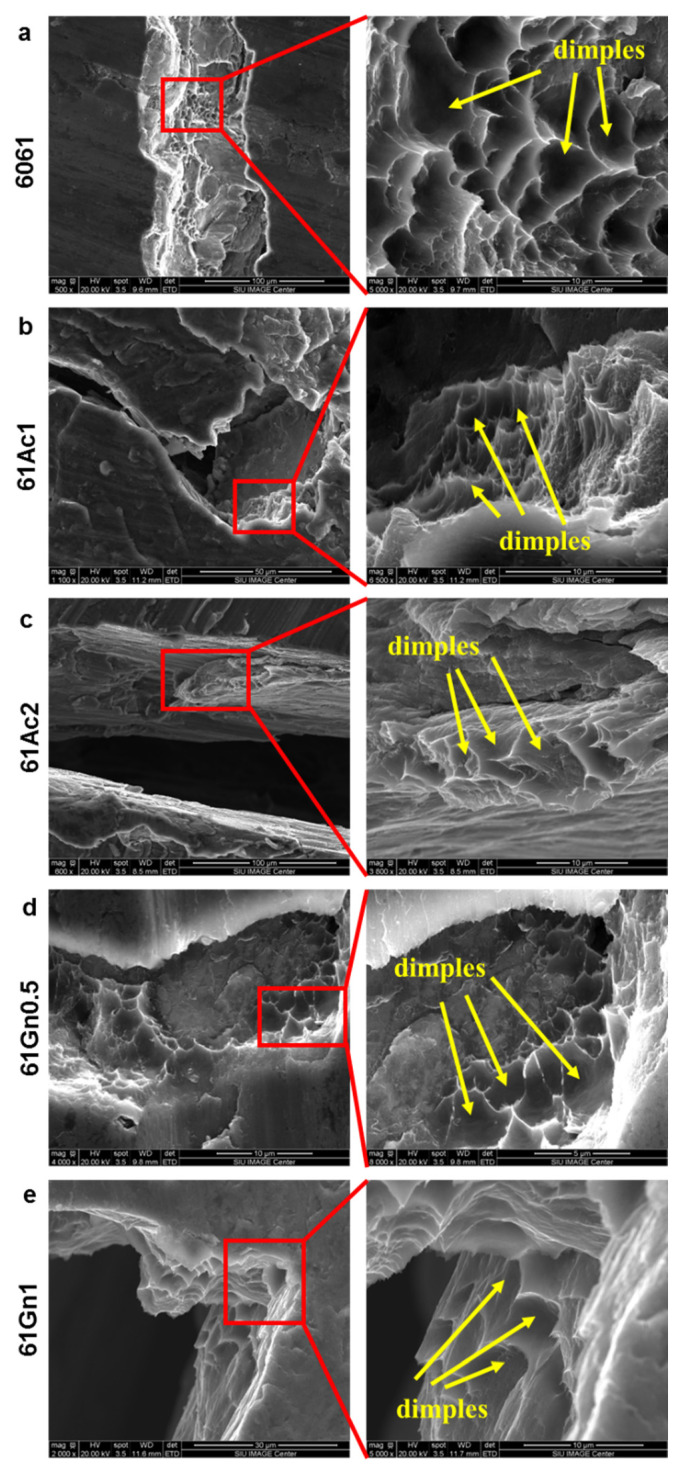
Fractured morphology of the nanocarbon 6061 AMCs, (**a**) 6061 (**b**) 61Ac1, (**c**) 61Ac2, (**d**) 61Gn0.5, and (**e**) 61Gn1.

Figure 7 shows the SEM images of the compression-fractured surfaces of the composites. They all presented dimples and ridges with regular morphology, as is typical for the elastic deformation and fracture of ductile materials [69]. These dimples also play a role as a nucleation site for the formation of voids and the propagation of cracks. However, the number of dimples and ridges was reduced as the amount of nanocarbon increased, showing the loss of the ductile nature in the composites, similar to the results shown elsewhere [70,71]. All the samples fractured at an angle of 45° with respect to the direction of the applied load, indicating that the fracture is a consequence of the shear forces involved in the load transfer from the matrix to the reinforcement [72].

## 4. Conclusions

Aluminum matrix composites reinforced with different volume fractions of activated nanocarbon and graphene nanoplatelets were fabricated by the powder metallurgy method. SEM micrographs presented a good distribution of carbon nanoparticles. After the milling procedure, a change in the original structure of the activated nanocarbon and graphene was detected with Raman spectroscopy. There was no evidence of the formation of intermediate phases, such as Al_4_C_3,_ which can affect the interaction between nanocarbon and aluminum. Mechanical properties were enhanced when compared to the unreinforced 6061 aluminum. Yield strength increased with both activated nanocarbon and graphene, being superior for the latter, but yield strength decreased with the increased amount of graphene reinforcement. Hardness presented a maximum increase of 26% for the sample with 2 vol. % of activated nanocarbon. Strengthening can be attributed to load transfer, dislocation strengthening, and grain refinement in hot-pressed composites. Following an initial decrease in elongation and modulus of toughness, they started to increase with the volume fraction of activated nanocarbon. The determination of densities also combined to elucidate the influence of reinforcement on the mechanical properties of the composites. Density Functional Theory (DFT) calculations for the phonon frequencies involved in the atomic displacements derived from carbon-aluminum interactions were concordant with the Raman analysis, which explained the results obtained for the experimental structural characterization.

## Figures and Tables

**Table 1:** Denomination of the samples prepared for this study.

Denomination	Description
6061	6061 aluminum without any reinforcement
61Ac1	6061 aluminum reinforced with 1 vol. % of activated nanocarbon
61Ac2	6061 aluminum reinforced with 2 vol. % of activated nanocarbon
61Gn0.5	6061 aluminum reinforced with 0.5 vol. % of graphene nanoplatelets
61Gn1	6061 aluminum reinforced with 1 vol. % of graphene nanoplatelets

## Data Availability

The data presented in this study are available on reasonable request from the corresponding author.

## References

[1] Pramanik A. (2016). Effects of reinforcement on wear resistance of aluminum matrix composites. Trans. Nonferrous Met. Soc. China.

[2] Mousavian R.T., Khosroshahi R.A., Yazdani S., Brabazon D., Boostani A.F. (2016). Fabrication of aluminum matrix composites reinforced with nano- to micrometer-sized SiC particles. Mater. Des..

[3] Liu Z.Y., Xu S.J., Xiao B.L., Xue P., Wang W.G., Ma Z.Y. (2012). Effect of ball-milling time on mechanical properties of carbon nanotubes reinforced aluminum matrix composites. Compos. Part A Appl. S..

[4] Guo B.S., Song M., Yi J.H., Ni S., Shen T., Du Y. (2017). Improving the mechanical properties of carbon nanotubes reinforced pure aluminum matrix composites by achieving non-equilibrium interface. Mater. Des..

[5] Rativa-Parada W., Nilufar S. (2023). Nanocarbon-Infused Metal Matrix Composites: A Review. JOM.

[6] Pan S.H., Wang T.L., Jin K.Y., Cai X.R. (2022). Understanding and designing metal matrix nanocomposites with high electrical conductivity: A review. J. Mater. Sci..

[7] Jiang Y.Y., Tan Z.Q., Xu R., Fan G.L., Xiong D.B., Guo Q., Su Y.S., Li Z.Q., Zhang D. (2018). Tailoring the structure and mechanical properties of graphene nanosheet/aluminum composites by flake powder metallurgy via shift-speed ball milling. Compos. Part A Appl. Sci. Manuf..

[8] Chak V., Chattopadhyay H., Dora T.L. (2021). Application of solid processing routes for the synthesis of graphene-aluminum composites—A review. Mater. Manuf. Process..

[9] Zhang L., Hou G.M., Zhai W., Ai Q., Feng J.K., Zhang L., Si P.C., Ci L.J. (2018). Aluminum/graphene composites with enhanced heat-dissipation properties by in-situ reduction of graphene oxide on aluminum particles. J. Alloys Compd..

[10] Yan S.J., Dai S.L., Zhang X.Y., Yang C., Hong Q.H., Chen J.Z., Lin Z.M. (2014). Investigating aluminum alloy reinforced by graphene nanoflakes. Mat. Sci. Eng. A.

[11] Xiong B.W., Liu K., Xiong W., Wu X., Sun J.Y. (2020). Strengthening effect induced by interfacial reaction in graphene nanoplatelets reinforced aluminum matrix composites. J. Alloys Compd..

[12] Zhang H.P., Xu C., Xiao W.L., Ameyama K., Ma C.L. (2016). Enhanced mechanical properties of Al5083 alloy with graphene nanoplates prepared by ball milling and hot extrusion. Mater. Sci. Eng. A.

[13] Saboori A., Novara C., Pavese M., Badini C., Giorgis F., Fino P. (2017). An Investigation on the Sinterability and the Compaction Behavior of Aluminum/Graphene Nanoplatelets (GNPs) Prepared by Powder Metallurgy. J. Mater. Eng. Perform..

[14] Zare R., Sharifi H., Saeri M.R., Tayebi M. (2019). Investigating the effect of SiC particles on the physical and thermal properties of Al6061/SiCp composite. J. Alloys Compd..

[15] Jeyasimman D., Sivaprasad K., Sivasankaran S., Ponalagusamy R., Narayanasamy R., Iyer V. (2015). Microstructural observation, consolidation and mechanical behaviour of AA 6061 nanocomposites reinforced by gamma-Al_2_O_3_ nanoparticles. Adv. Powder Technol..

[16] Zheng T.Q., Pan S.H., Liu J.K., Moodispaw M., Luo A.A., Taub A.I., Li X.C. (2023). Study on nano-treating of Al-Mg-Si-Cu alloys with TiC nanoparticles. J. Alloys Compd..

[17] Fuse K., Badheka V., Patel V., Andersson J. (2021). Dual sided composite formation in Al 6061/B4C using novel bobbin tool friction stir processing. J. Mater. Res. Technol..

[18] Desai A.M., Paul T.R., Mallik M. (2020). Mechanical properties and wear behavior of fly ash particle reinforced Al matrix composites. Mater. Res. Express.

[19] Pazhouhanfar Y., Eghbali B. (2018). Microstructural characterization and mechanical properties of TiB2 reinforced Al6061 matrix composites produced using stir casting process. Mater. Sci. Eng. A.

[20] Chamroune N., Mereib D., Delange F., Caillault N., Lu Y.F., Grosseau-Poussard J.L., Silvain J.F. (2018). Effect of flake powder metallurgy on thermal conductivity of graphite flakes reinforced aluminum matrix composites. J. Mater. Sci..

[21] Huang Y., Ouyang Q.B., Zhang D., Zhu J., Li R.X., Yu H. (2014). Carbon Materials Reinforced Aluminum Composites: A Review. Acta Metall. Sin. Engl..

[22] Mohammed S.M.A.K., Chen D.L.L. (2020). Carbon Nanotube-Reinforced Aluminum Matrix Composites. Adv. Eng. Mater..

[23] Kumar P.A., Namboodiri V.V., Joshi G., Mehta K.P. (2021). Fabrication and applications of fullerene-based metal nanocomposites: A review. J. Mater. Res..

[24] Su J.L., Teng J. (2021). Recent progress in graphene-reinforced aluminum matrix composites. Front. Mater. Sci..

[25] Akinwande D., Brennan C.J., Bunch J.S., Egberts P., Felts J.R., Gao H.J., Huang R., Kim J.S., Li T., Li Y. (2017). A review on mechanics and mechanical properties of 2D materials-Graphene and beyond. Extreme Mech. Lett..

[26] Papageorgiou D.G., Kinloch I.A., Young R.J. (2017). Mechanical properties of graphene and graphene-based nanocomposites. Prog. Mater. Sci..

[27] Vicarelli L., Heerema S.J., Dekker C., Zandbergen H.W. (2015). Controlling Defects in Graphene for Optimizing the Electrical Properties of Graphene Nanodevices. ACS Nano.

[28] Pop E., Varshney V., Roy A.K. (2012). Thermal properties of graphene: Fundamentals and applications. MRS Bull..

[29] Tiwari S.K., Sahoo S., Wang N., Huczko A. (2020). Graphene research and their outputs: Status and prospect. J. Sci. Adv. Mater. Dev..

[30] Xie Y.M., Meng X.C., Mao D.X., Qin Z.W., Wan L., Huang Y.X. (2021). Homogeneously Dispersed Graphene Nanoplatelets as Long-Term Corrosion Inhibitors for Aluminum Matrix Composites. ACS Appl. Mater. Interfaces.

[31] Chen F., Gupta N., Behera R.K., Rohatgi P.K. (2018). Graphene-Reinforced Aluminum Matrix Composites: A Review of Synthesis Methods and Properties. JOM.

[32] Fleker O., Borenstein A., Lavi R., Benisvy L., Ruthstein S., Aurbach D. (2016). Preparation and Properties of Metal Organic Framework/Activated Carbon Composite Materials. Langmuir.

[33] Lu L., Lai M.O., Yeo J.L. (1999). In situ synthesis of TiC composite for structural application. Compos. Struct..

[34] Ge X., Klingshirn C., Morales M., Wuttig M., Rabin O., Zhang S., Salamanca-Riba L.G. (2021). Electrical and structural characterization of nano-carbon–aluminum composites fabricated by electro-charging-assisted process. Carbon.

[35] Eisay A.M.S., Turkyilmaz A. (2021). Production of Aluminum Matrix Composite Material by Active Carbon Additive. J. Inorg. Organomet. Polym. Mater..

[36] Jaim H.I., Isaacs R.A., Rashkeev S.N., Kuklja M., Cole D.P., LeMieux M.C., Jasiuk I., Nilufar S., Salamanca-Riba L.G. (2016). Sp2 carbon embedded in Al-6061 and Al-7075 alloys in the form of crystalline graphene nanoribbons. Carbon.

[37] Giannozzi P., Baroni S., Bonini N., Calandra M., Car R., Cavazzoni C., Ceresoli D., Chiarotti G.L., Cococcioni M., Dabo I. (2009). QUANTUM ESPRESSO: A modular and open-source software project for quantum simulations of materials. J. Phys. Condens. Matter..

[38] (2022). Standard Test Method for Knoop and Vickers Hardness of Materials.

[39] (2018). Standard Test Methods of Compression Testing of Metallic Materials at Room Temperature.

[40] Gao X., Yue H.Y., Guo E.J., Zhang H., Lin X.Y., Yao L.H., Wang B. (2016). Preparation and tensile properties of homogeneously dispersed graphene reinforced aluminum matrix composites. Mater. Des..

[41] Tabandeh-Khorshid M., Kumar A., Omrani E., Kim C., Rohatgi P. (2020). Synthesis, characterization, and properties of graphene reinforced metal-matrix nanocomposites. Compos. B Eng..

[42] Bisht A., Srivastava M., Kumar R.M., Lahiri I., Lahiri D. (2017). Strengthening mechanism in graphene nanoplatelets reinforced aluminum composite fabricated through spark plasma sintering. Mater. Sci. Eng. A.

[43] Hu Z.R., Chen F., Xu J.L., Nian Q., Lin D., Chen C.J., Zhu X., Chen Y., Zhang M. (2018). 3D printing graphene-aluminum nanocomposites. J. Alloys Compd..

[44] Popov M., Medvedev V., Blank V., Denisov V., Kirichenko A., Tat’yanin E., Aksenenkov V., Perfilov S., Lomakin R., D’yakov E. (2010). Fulleride of aluminum nanoclusters. J. Appl. Phys..

[45] Perez-Bustamante R., Bolanos-Morales D., Bonilla-Martinez J., Estrada-Guel I., Martinez-Sanchez R. (2014). Microstructural and hardness behavior of graphene-nanoplatelets/aluminum composites synthesized by mechanical alloying. J. Alloys Compd..

[46] Bastwros M., Kim G.Y., Zhu C., Zhang K., Wang S.R., Tang X.D., Wang X.W. (2014). Effect of ball milling on graphene reinforced Al6061 composite fabricated by semi-solid sintering. Compos. B Eng..

[47] Cancado L.G., Takai K., Enoki T., Endo M., Kim Y.A., Mizusaki H., Jorio A., Coelho L.N., Magalhaes-Paniago R., Pimenta M.A. (2006). General equation for the determination of the crystallite size L-a of nanographite by Raman spectroscopy. Appl. Phys. Lett..

[48] Sirikumara H.I., Rativa-Parada W., Karunanithy R., Sivakumar P., Nilufar S., Jayasekera T. (2022). Atomic composition/configuration dependent bulk moduli of Al-C composites. AIP Adv..

[49] Pan S.H., Yuan J., Zheng T.Q., She Z.Y., Li X.C. (2021). Interfacial thermal conductance of in situ aluminum-matrix nanocomposites. J. Mater. Sci..

[50] Shin S.E., Bae D.H. (2015). Deformation behavior of aluminum alloy matrix composites reinforced with few-layer graphene. Compos. Part A Appl. Sci. Manuf..

[51] Wang J.Y., Li Z.Q., Fan G.L., Pan H.H., Chen Z.X., Zhang D. (2012). Reinforcement with graphene nanosheets in aluminum matrix composites. Scr. Mater..

[52] Rashad M., Pan F.S., Tang A.T., Asif M. (2014). Effect of Graphene Nanoplatelets addition on mechanical properties of pure aluminum using a semi-powder method. Prog. Nat. Sci. Mater..

[53] Ramadan S., Taha M.A., El-Meligy W.M., Saudi H.A., Zawrah M.F. (2023). Influence of Graphene Content on Sinterability and Physico-Mechanical Characteristics of Al/Graphene Composites Prepared via Powder Metallurgy. Biointerface Res. Appl. Chem..

[54] Gurbuz M., Senel M.C., Koc E. (2018). The effect of sintering time, temperature, and graphene addition on the hardness and microstructure of aluminum composites. J. Compos. Mater..

[55] Huang C.Y., Hu S.P., Chen K. (2019). Influence of rolling temperature on the interfaces and mechanical performance of graphene-reinforced aluminum-matrix composites. Int. J. Miner. Met. Mater..

[56] Lazarova R., Mourjeva Y., Petkov V., Anestiev L., Marinov M., Dimitrova R., Shuleva D. (2022). Microstructure and Mechanical Properties of Aluminum: Graphene Composites Produced by Powder Metallurgical Method. J. Mater. Eng. Perform..

[57] Turan M.E. (2019). Investigation of mechanical properties of carbonaceous (MWCNT, GNPs and C60) reinforced hot-extruded aluminum matrix composites. J. Alloys Compd..

[58] Shin S.E., Choi H.J., Shin J.H., Bae D.H. (2015). Strengthening behavior of few-layered graphene/aluminum composites. Carbon.

[59] Tian W.-M., Li S.-M., Wang B., Chen X., Liu J.-H., Yu M. (2016). Graphene-reinforced aluminum matrix composites prepared by spark plasma sintering. Int. J. Miner. Metall. Mater..

[60] El-Ghazaly A., Anis G., Salem H.G. (2017). Effect of graphene addition on the mechanical and tribological behavior of nanostructured AA2124 self-lubricating metal matrix composite. Compos. Part A Appl. Sci. Manuf..

[61] Garg P., Jamwal A., Kumar D., Sadasivuni K.K., Hussain C.M., Gupta P. (2019). Advance research progresses in aluminium matrix composites: Manufacturing & applications. J. Mater. Res. Technol..

[62] Zeng M., Chen H.M., Tao X.M., Ouyang Y.F. (2023). Mechanical Property and Corrosion Behavior of Powder-Metallurgy-Processed 3D Graphene-Networks-Reinforced Al Matrix Composites. Crystals.

[63] Singh J., Chauhan A. (2016). Characterization of hybrid aluminum matrix composites for advanced applications—A review. J. Mater. Res. Technol..

[64] Cavaliere P., Sadeghi B., Shabani A. (2017). Carbon nanotube reinforced aluminum matrix composites produced by spark plasma sintering. J. Mater. Sci..

[65] Zare H., Jahedi M., Toroghinejad M.R., Meratian M., Knezevic M. (2016). Microstructure and mechanical properties of carbon nanotubes reinforced aluminum matrix composites synthesized via equal-channel angular pressing. Mater. Sci. Eng. A.

[66] Khan M., Amjad M., Khan A., Ud-Din R., Ahmad I., Subhani T. (2017). Microstructural evolution, mechanical profile, and fracture morphology of aluminum matrix composites containing graphene nanoplatelets. J. Mater. Res..

[67] El-Daly A.A., Abdelhameed M., Hashish M., Daoush W.M. (2013). Fabrication of silicon carbide reinforced aluminum matrix nanocomposites and characterization of its mechanical properties using non-destructive technique. Mater. Sci. Eng. A.

[68] Kumar K.R., Kiran K., Sreebalaji V.S. (2017). Micro structural characteristics and mechanical behaviour of aluminium matrix composites reinforced with titanium carbide. J. Alloys Compd..

[69] Xiong B.W., Liu K., Yan Q.S., Xiong W., Wu X. (2020). Microstructure and mechanical properties of graphene nanoplatelets reinforced Al matrix composites fabricated by spark plasma sintering. J. Alloys Compd..

[70] Liu Q., Ke L.M., Liu F.C., Huang C.P., Xing L. (2013). Microstructure and mechanical property of multi-walled carbon nanotubes reinforced aluminum matrix composites fabricated by friction stir processing. Mater. Des..

[71] Lou S.M., Li Y.M., Cheng B.J., Ran L.W., Bai X.F., Chen P., Wang Q.B. (2023). Microstructural and Mechanical Properties of Longitudinal Welds in Porthole Die Extrudates of a 0.5 wt.% GNP/Al Composite. Metals.

[72] Shin S.E., Choi H.J., Hwang J.Y., Bae D.H. (2015). Strengthening behavior of carbon/metal nanocomposites. Sci. Rep..

